# RNA Sequencing Reveals Differential Expression of Mitochondrial and Oxidation Reduction Genes in the Long-Lived Naked Mole-Rat When Compared to Mice

**DOI:** 10.1371/journal.pone.0026729

**Published:** 2011-11-03

**Authors:** Chuanfei Yu, Yang Li, Andrew Holmes, Karol Szafranski, Chris G. Faulkes, Clive W. Coen, Rochelle Buffenstein, Matthias Platzer, João Pedro de Magalhães, George M. Church

**Affiliations:** 1 Department of Genetics, Harvard Medical School, Boston, Massachusetts, United States of America; 2 Integrative Genomics of Ageing Group, Institute of Integrative Biology, University of Liverpool, Liverpool, United Kingdom; 3 Fritz Lipmann Institute – Leibniz Institute for Age Research, Jena, Germany; 4 School of Biological and Chemical Sciences, Queen Mary University of London, London, United Kingdom; 5 Reproductive Neurobiology and Early Life Origins of Disease, Division of Women's Health, King's College London, London, United Kingdom; 6 Barshop Institute for Aging and Longevity Studies and Department of Physiology, University of Texas Health Science Center at San Antonio, San Antonio, Texas, United States of America; University of Washington, United States of America

## Abstract

The naked mole-rat (*Heterocephalus glaber*) is a long-lived, cancer resistant rodent and there is a great interest in identifying the adaptations responsible for these and other of its unique traits. We employed RNA sequencing to compare liver gene expression profiles between naked mole-rats and wild-derived mice. Our results indicate that genes associated with oxidoreduction and mitochondria were expressed at higher relative levels in naked mole-rats. The largest effect is nearly 300-fold higher expression of epithelial cell adhesion molecule (Epcam), a tumour-associated protein. Also of interest are the protease inhibitor, alpha2-macroglobulin (A2m), and the mitochondrial complex II subunit Sdhc, both ageing-related genes found strongly over-expressed in the naked mole-rat. These results hint at possible candidates for specifying species differences in ageing and cancer, and in particular suggest complex alterations in mitochondrial and oxidation reduction pathways in the naked mole-rat. Our differential gene expression analysis obviated the need for a reference naked mole-rat genome by employing a combination of Illumina/Solexa and 454 platforms for transcriptome sequencing and assembling transcriptome contigs of the non-sequenced species. Overall, our work provides new research foci and methods for studying the naked mole-rat's fascinating characteristics.

## Introduction

Understanding the mechanisms underlying the exceptional resistance to ageing processes and age-related diseases of long-lived species may lead to the discovery of senescence-retarding mechanisms [1]. Recent work using the naked mole-rat (*Heterocephalus glaber*, Ruppell 1842) as a model for successful ageing has a great potential to provide clues about species differences in ageing. *Heterocephalus* belongs to the family Bathyergidae (African mole-rats) that includes 6 genera and possibly more than 50 other species. They are distinct from laboratory mice and rats being part of the Hystricognath infraorder of rodents that includes the South American Caviomorphs (e.g. guinea pigs), and African Phiomorphs such as porcupines, rock rats and cane rats [2], [3], [4]. The naked mole-rat is the longest-lived rodent known with a lifespan of over 30 years [5]. These animals appear to be cancer resistant since neither tumors nor lesions have been observed to date during necropsy [6], which is in marked contrast to mice since even long-lived wild-derived animals of that species die primarily of neoplasia [7]. There is an immense interest in identifying the specific adaptations responsible for the unique traits of the naked mole-rat, and particularly their longevity and cancer resistance.

The natural habitat of the naked mole-rat as well as its eusocial behaviour and phylogenetic history may be among the reasons why naked mole-rats have evolved such an extreme longevity compared to other rodents. Many of the hystricognath rodents are long-lived (e.g., porcupine, guinea pig, other mole-rats) when compared with other rodent clades. Furthermore, extended longevity is also commonly associated with group living [8] as it enhances inclusive fitness by kinship, cooperative care of young and intergenerational transfer of information [6]. Naked mole-rats live underground and it is well-known that subterranean animals are protected from both climatic extremes and predation, which lowers their extrinsic mortality rate and favours the evolution of a long lifespan [6], [9], [10].

In contrast to other rodents, such as mice and rats which normally do not live more than 4 years, naked mole-rats show small age-related changes in morphology and maintain reproductive potential as well as physiological function and activity at old ages [5]. They also exhibit small age-related changes in mitochondrial mass and efficiency [11], antioxidant activity [12], membrane composition [13] and lipid peroxidation [14]. Compared to mice, young naked mole-rats show high levels of oxidative stress to lipids, proteins and DNA [15]. However, they show attenuated age-related changes in protein oxidation and resistance to protein unfolding in the liver, suggesting that protein stability, turnover and resistance to the accumulation of oxidative damage may be important players in the naked mole-rats' extreme longevity when compared to mice [16].

It has long been argued that differences in gene expression may underlie species phenotypic differences [17], including species differences in ageing and longevity [18]. One previous gene expression study in liver found evidence that FOXO1, which has a role in the regulation of metabolism and longevity, is up-regulated in humans when compared to chimpanzees, which the authors suggest could reflect longevity differences between these species [19]. Given the marked phenotypic variance between the naked mole-rats and other rodents, we were interested in finding the differences in the mRNA composition of naked mole-rats that may be able to explain, at least partially, their cancer-resistance and extreme longevity. By employing 2^nd^-generation sequencing technologies we were able to compare liver gene expression profiles of naked mole-rats and mice and identify genes over-expressed in naked mole-rats, some of which have previously been related to ageing.

## Results

We compared liver gene expression profiles from naked mole-rats and wild-derived mice. Because our focus is on species comparisons, we used wild-derived mice as these are expected to be more representative of *Mus musculus* than laboratory strains and are, in fact, longer-lived [7], [20]. Samples from young adult male animals were employed in our analyses. RNA was extracted, triplicate samples were pooled, libraries prepared according to established protocols and sequenced using the Illumina/Solexa platform (see Materials and Methods). Libraries were sequenced twice, once with 39 bp Solexa single-end reads and again with 76 bp Solexa single-end reads. We then established a method to map naked mole-rat reads to their mouse genes orthologs. Briefly, we obtained an assembly of the naked mole-rat transcriptome generated by 454 sequencing and complemented by Illumina/Solexa resulting in 77,086 contigs. We then mapped 21.2 M naked mole-rat single end 76 bp and 9.2 M single end 39 bp Solexa reads to the contigs of which 59.1% 76 bp Solexa reads were mapped successfully while only around 36.5% of the 39 bp Solexa reads were mapped successfully (see Materials and Methods). To map naked mole-rat reads to mouse genes, we employed BLAST to map the contigs of the assembly onto mouse cDNAs and kept roughly 51% of contigs mapping unambiguously. Ultimately, out of the 12.2 M 76 bp reads with good mapping quality, 9.0 M could be assigned to an orthologous mouse gene.

After the above steps, we obtained a list of genes with their read numbers for the naked mole-rat Solexa data. We then constructed a reference library for mouse consisting of the 3′ end of all mouse cDNA transcripts. The wild-derived mouse Solexa reads were then mapped to this reference library with 13.2 M out of the initial 24.3 M mouse 76 bp Solexa reads mapping successfully, while 8.8 M out of these 13.2 M reads could be mapped to a mouse transcript with a naked mole-rat contig ortholog. Since two sequencing runs can have a different number of reads output and two samples can have different bias depending on how a library is prepared before sequencing, we verified for systematic biases and found that the distribution of log differential expression was as expected and no normalization was judged to be necessary (see Materials and Methods).

### Top over-expressed genes in naked mole-rats

Because of the larger number of reads and higher mapping accuracy of 76 bp Solexa reads, the corrected fold difference between naked mole-rat and mice for each gene was computed using the 76 bp data with 100 pseudo-counts added to both naked mole-rat and mouse read counts. The 39 bp data were used as replicates to determine how reliable the computed fold difference is. For example, Epcam (rank 1) and Eif4g1 (rank 5) had relatively low 39 bp read counts. Because the coverage of the naked mole-rat transcriptome is imperfect, under-expressed genes in the naked mole-rat may be false positives (e.g. unmapped naked mole-rat reads). Moreover, the number of mouse genes with at least one read in our mouse dataset (17,446) is greater than the number of mouse genes with at least one read in the naked mole-rat dataset (11,198) and thus we focused our analysis on over-expressed genes in the naked mole-rat.

We obtained 660 genes over-expressed 5-fold or more, 268 genes 10-fold or more and 153 genes 15-fold or more (Dataset S1). The top over-expressed genes in the naked mole-rat can be found in Table 1 with Epcam as the top gene and A2m as the only gene previously associated with ageing. Among the top 100 genes, Igf2 and Sdhc were the other ageing-associated genes (Dataset S2). Our full results are available online (http://www.naked-mole-rat.org/gene_expression.php).

**Table 1:** Genes with highest fold expression differences between naked mole-rats (NMRs) and wild-derived mice. The ratio is computed with 100 pseudo-counts added to both 76 bp counts.

	Corrected Fold difference	NMR count		Mouse count		Gene symbol	Gene name
		76 bp	39 bp	76 bp	39 bp		
1	290.4	37073	596	27	4	Epcam	Epithelial cell adhesion molecule
2	252.9	31261	1075	23	14	Suclg2	Succinyl-CoA ligase
3	207.7	25654	926	23	1	Gulp1	PTB domain-containing engulfment adapter protein 1
4	189.8	31590	1166	66	3	Acmsd	2-amino-3-carboxymuconate-6-semialdehyde decarboxylase
5	183.1	19309	339	5	8	Eif4g1	Eukaryotic translation initiation factor 4 gamma 1
6	169.9	18421	4336	8	0	Tyro3	Tyrosine-protein kinase receptor TYRO3 Precursor
7	154.2	19335	2868	25	21	Upb1	Beta-ureidopropionase
8	146.5	14695	903	0	0	Tspan1	Tetraspanin 1
9	143.9	202485	14461	1307	0	A2m	Alpha-2-macroglobulin-P Precursor
10	135.8	21227	981	56	168	Ugt1a10	UDP-glucuronosyltransferase 1–7C Precursor
11	131.4	14613	2961	11	7	Scd3	Stearoyl-coenzyme A desaturase 3
12	123.6	12512	4230	1	0	Rpl39l	Ribosomal protein L39-like protein
13	122.5	74402	3182	507	144	Rpl26	60S ribosomal protein L26
14	112.5	21058	7205	87	358	Fau	Ubiquitin-like protein FUBI
15	107.7	11959	721	11	7	Amacr	Alpha-methylacyl-CoA racemase
16	104.6	19454	5099	86	4	Crym	Mu-crystallin homolog
17	95.7	11671	1140	22	6	Upp2	Uridine phosphorylase 2
18	90.8	9523	1438	5	0	Moxd1	DBH-like monooxygenase protein 1 Precursor
19	90.5	16106	3522	78	12	Mt2	Metallothionein-2
20	88.3	13769	1218	56	56	Sdc2	Syndecan-2 Precursor

### Functional analysis of genes over-expressed in naked mole-rats

To identify pathways and biological functions that tend to be over-expressed in the naked mole-rat, we used the Database for Annotation, Visualization and Integrated Discovery (DAVID) to identify functional categories associated with more over-expressed genes than expected by chance [21]. Using DAVID and setting all mouse genes for which an unambiguous naked mole-rat orthologous contig exists as background, we found that genes over-expressed 5-fold or more in the naked mole-rat are enriched in genes associated with mitochondrion (p = 8.1E-7), acetylation (1.1E-6) and oxidoreductase (4.7E-6); see Table 2 for top hits and Dataset S3 for full results. We also found genes associated with mitochondrial matrix (2.1E-6) and oxidation reduction (6.7E-6) enriched in our hits when using GO terms. For the KEGG pathways analysis, we found fatty acid metabolism (4.0E-3) and butanoate metabolism (7.0E-3).

**Table 2:** Top non-redundant categories among genes over-expressed 5-fold or more in naked mole-rats when compared to wild-derived mice as derived from DAVID.

Term	Count	P-value	Benjamini
Functional categories			
Mitochondrion	66	8.1E-07	3.4E-04
Acetylation	150	1.1E-06	2.4E-04
Oxidoreductase	44	4.7E-06	6.5E-04
Transit peptide	44	7.2E-06	7.5E-04
NAD	17	1.3E-03	0.11
Endoplasmic reticulum	46	1.4E-03	0.095
GO categories			
Mitochondrial matrix (GO:0005759)	25	2.1E-06	7.0E-04
Oxidation reduction (GO:0055114)	50	6.7E-06	0.013
KEGG pathways			
Fatty acid metabolism (mmu00071)	8	4.0E-03	0.45
Butanoate metabolism (mmu00650)	7	7.0E-03	0.41

To confirm the above results we repeated the DAVID analysis using genes 10-fold or more and 15-fold or more over-expressed in the naked mole-rat. We found very similar results where acetylation and mitochondrion were the most enriched functional categories in, respectively, 10-fold and 15-fold cutoffs and butanoate metabolism and fatty acid metabolism were found to be the most enriched in our pathway analysis (see Dataset S3). A few categories with higher p-values also caught our attention such as lipid biosynthetic process (3.5E-3; 5-fold), endoplasmic reticulum (1.4E-03; 5-fold), ER-nuclear signaling pathway (6.0E-03; 5-fold), response to oxidative stress (2.2E-02; 5-fold), protein repair (3.5E-02; 15-fold) and metabolism of xenobiotics by cytochrome P450 (5.4E-02; 15-fold).

### Validation of results using microarray data and qRT-PCR

**Figure 1:**
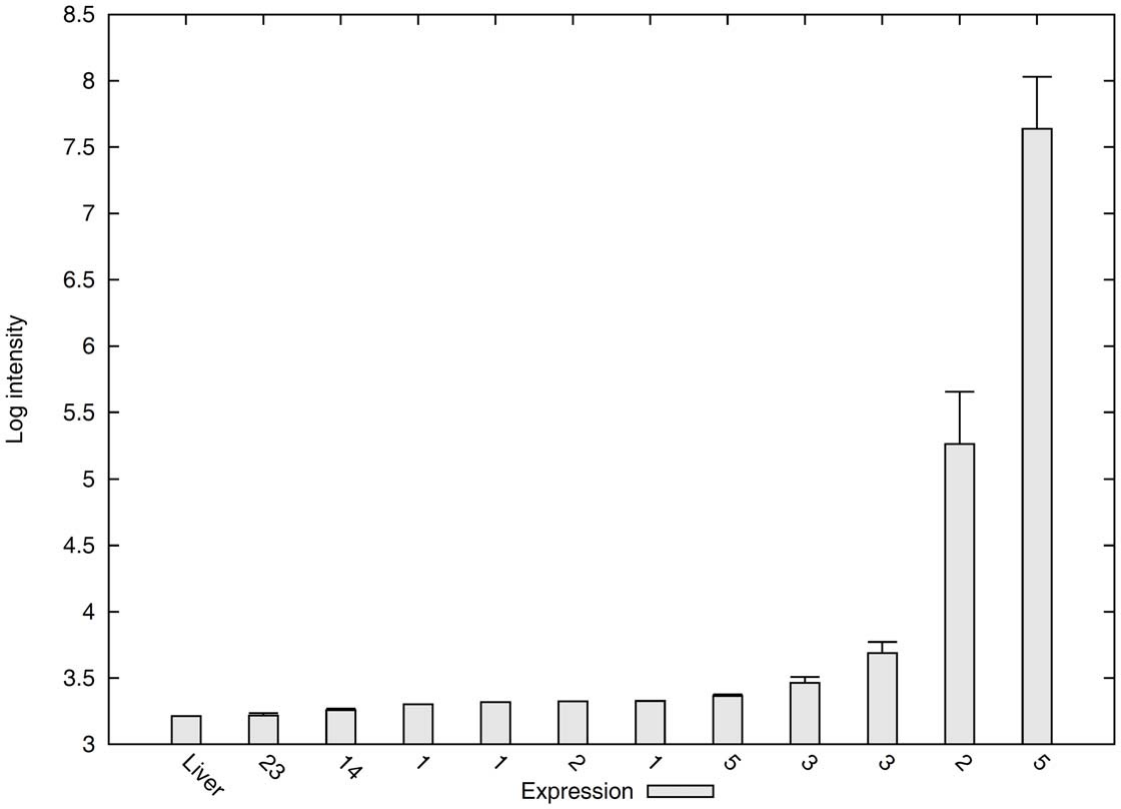
A2m expression in mouse liver compared to other tissues. The number under each bin is the number of tissues with similar expression level, while the bars on each bin denote the standard deviation of the tissues gene expression in each bin.

To validate some of the most interesting candidates found in our study, we retrieved expression data from the mouse expression database GeneAtlas (http://www.geneatlas.org/), which contains microarray intensity of gene expression in many different tissues. For genes over-expressed in the naked mole-rat, we plotted the histogram of liver absolute expression against the gene expression in other tissues. We expected to find low absolute expression of genes with low Solexa read counts. As shown in Figure 1, the *A2m* expression is low in liver which corroborates the low *A2m* read count we found in our Solexa sequencing results.

**Figure 2:**
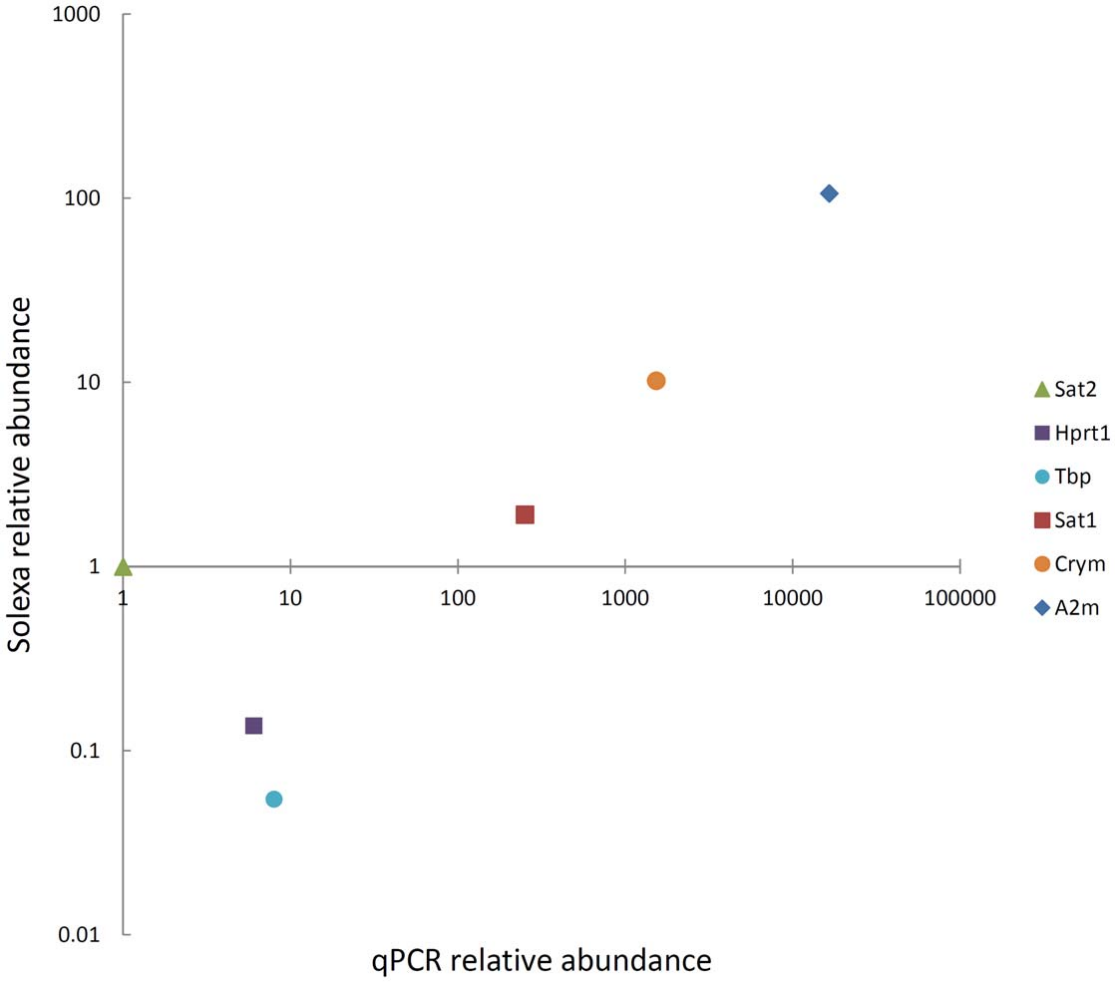
Comparison of transcript abundance between Solexa and qPCR data. Abundances of *A2m*, *Sat1*, *Sat2*, *Hprt1*, *Tbp*, and *Crym* transcripts relative to *Sat2* generated from Solexa data and qPCR analyses are shown.

In order to validate the transcript abundances of over-expressed genes identified by the Solexa mapping, quantitative real-time PCR (qPCR) was employed to analyse gene expression. Six transcripts were investigated, including three identified as strongly over-expressed (*A2m*, *Sat2* and *Crym*), one mildly over-expressed (*Sat1*) and two housekeeping transcripts (*Hprt1* and *Tbp*). Solexa reads, and qPCR Ct values for all transcripts were calibrated to those of *Sat2*, which had the lowest mean Ct value to generate relative transcript abundances. The transcripts *A2m Crym*, and *Sat1* found to be most abundant from the Solexa analysis were similarly found to be the most abundant in the qPCR data, although qPCR generated higher transcript abundances compared to Solexa (Figure 2). Transcripts from genes determined to be of a relatively lower abundance (*Sat2*, *Hprt1* and *Tbp*) from Solexa data also showed a lower relative abundance in the qPCR analysis. In the cases of *Hprt1* and *Tbp*, only few Solexa reads mapped (249 and 57 respectively), but expression determined via qPCR was unexpectedly high which could reflect the above-mentioned concerns about under-expressed genes and why we focused on over-expressed genes.

## Discussion

The naked mole-rat is not only the longest-lived rodent, but has a much longer lifespan than expected for its relatively small body size and has been shown to be extremely resistant to neoplasia. Furthermore, since a number of other rodents including mice, rats and guinea pigs already have had their genome sequenced, the naked mole-rat is a prime candidate for comparative genomics studies. In this study, we compared the expression of genes between naked mole-rats and mice by directly sequencing cDNA libraries constructed from liver samples with the aim to gather insights regarding the molecular mechanisms responsible for the naked mole-rat adaptations as well as develop methods for functional genomics in this exotic species.

Differential expression analyses using 2^nd^-generation sequencing platforms such as Illumina/Solexa, ABI SOLiD have increased in popularity due to their low cost and high throughput [22]. However, due to the short lengths of their read output, a reference genome is also needed and has limited the number of 2^nd^-generation transcriptome studies in non-sequenced species. In our study, we show that it is possible to conduct differential gene expression analyses in non-sequenced mammalian species using only 2^nd^-generation sequencing technologies by assembling transcriptome contigs of the non-sequenced species. Using a combination of 2^nd^-generation sequencing platforms (Roche 454 and Illumina/Solexa), we were able to compare gene expression between wild-derived mice and naked mole-rats without using a naked mole-rat reference genome. Our approach and methods not employing a reference genome may be applicable to other studies of non-traditional mammalian model organisms.

One caveat of our method is that only genes over-expressed in the naked mole-rat when compared to mice can be accurately identified. Genes detected to be under-expressed in the naked mole-rat by our approach may be real biological phenomena or may be due to a lack of coverage from our assembly of the naked mole-rat transcriptome. Under-expressed ageing-related genes (with over 1,000 reads in the naked mole-rat and hence with some coverage) that caught our attention and may be subject of further studies included *Gpx1*, *Gpx4* and *Rgn* (Dataset S2). Another study recently found lower levels of *Gpx1* in naked mole-rat liver than in mouse liver [23], so some of our under-expressed genes may be phenotypically relevant. Due to the incomplete naked mole-rat transcriptome and annotations, however, it may be that Solexa read counts for some transcripts were extremely low due to poor mapping, rather than low expression. This may also explain why higher relative abundances of *Hprt1* and *Tbp* were observed in the qPCR analysis when compared to the Solexa data. Nonetheless, our analyses show that, unlike in mice, naked mole-rat *A2m*, *Crym*, and *Sat2* are expressed in the liver and demonstrate the capacity of our method to detect over-expressed genes.

We found a considerable number of genes over-expressed in the naked mole-rat, many with large expression changes. One possibility is that a shift in tissue-specificity may account for some of the dramatic over-expression levels we found so that a gene with negligible expression in the mouse liver, but some expression in the naked mole-rat's, will be detected as considerably over-expressed in the naked mole-rat liver. Another other hypothesis is that some genes evolved different functions in the naked mole-rat liver.

### Candidate over-expressed genes in the naked mole-rat associated with its adaptations

In addition to longevity, mice and naked mole-rats diverge on many other physiological traits and live in extremely different natural habitats. It is therefore impossible to tell for certain whether an over-expressed gene or pathway in the naked mole-rat is responsible for its increased longevity or other phenotypic traits well suited to a subterranean milieu. In spite of this caveat, we identified genes and pathways over-expressed in the naked mole-rat that have been previously associated with ageing and therefore may provide clues for understanding the differences in longevity between these species. These may also be candidates for further functional studies. One of these genes is stearoyl-coenzyme A desaturase (Scd3) which has been detected as the 11^th^ most over-expressed gene in the naked mole-rat (14,613 76 bp reads vs 11; 2961 39 bp reads vs 7). Scd3 is a fatty acid desaturase involved in lipid biosynthetic, lipid metabolic and oxidation-reduction processes which, as discussed later, all have previously been associated with ageing.

One of the most interesting over-expressed genes in the naked mole-rat is the serum pan-protease inhibitor, alpha2-macroglobulin (A2m). While A2m is expressed at a low level in wild-derived mouse liver (1,307 reads out of 8.8 M), we detected almost 202,500 (out of 9.0 M) reads in naked mole-rat liver which makes it among the top 20 most over-expressed genes. Interestingly, A2m is listed as a candidate protein relevant to the human ageing process in GenAge, a database of ageing- and longevity-associated genes [24]. A2m is also known to interact with longevity-associated ApoE (apolipoprotein E) and is associated with Alzheimer's disease [25]. Furthermore, A2m was determined to be a biomarker for ageing in vivo as its mRNA expression level showed positive correlation with age [26]. The function of A2m as a proteinase inhibitor may also be of interest in the context of protein turnover regulation in naked mole-rats, which Pérez et al. (2009) argue to be a key contributor to their extreme longevity.

Another gene that caught our attention is *Epcam*, also known as *Tacstd1*, which is at the top of our list with 37,073 76 bp reads in NMR and only 27 in mouse (596 39 bp NMR vs 4 mouse reads). Epcam is a transmembrane glycoprotein hypothesized to function as a cell adhesion molecule which interferes with cadherin-mediated cell–cell contact [27]. Furthermore, Epcam has been shown to promote cell cycling and enhance proliferation [28] and it is found to be over-expressed on epithelial progenitors, carcinomas and cancer-initiating cells [29]. While we cannot explain the over-expression of a tumour-associated gene in the cancer-resistant naked mole-rat, we suggest Epcam is a promising target for future studies. In the context of cancer, another strongly over-expressed gene (3^rd^ in our list) in the naked mole-rat of potential interest is *Gulp1*, an adaptor protein that promotes phagocytosis of apoptotic cells [30], and might therefore contribute to species differences in tissue turnover. Lastly, one gene recently hypothesized to be important in species longevity is Nrf2 [31], also known as Nfe2l2, which we interestingly found over 6-fold over-expressed in the naked mole-rat (Dataset S1). All in all, our findings warrant further studies of a number of genes (our full results are available in Datasets S1 and S2) and open new research avenues.

### Candidate pathways for extreme longevity divergence in the naked mole-rat

Within over-expressed genes in the naked mole-rat, genes associated with oxidoreduction were strongly overrepresented as well as genes associated with mitochondria and more specifically mitochondrial matrix. Consistent with the free radical theory of ageing, the over-expression of genes related to oxidoreduction could protect the naked mole-rat from reactive oxygen species. Indeed, the succinate dehydrogenase cytochrome b560 subunit (Sdhc), a member of the mitochondrial electron transport chain, was one of the few ageing-related genes strongly over-expressed in the naked mole-rat. However, the naked mole-rat exhibits greater levels of oxidative damage in lipids, DNA and proteins [15], thus the over-expression of the oxidoreduction and mitochondria genes could reflect a higher need for ROS protection and/or the cause of those higher levels. Given that naked mole-rats have a lower body temperature than mice [32], in addition to living in the aforementioned hypoxic environment, differential expression of genes related to metabolism and energy may be due to other physiological differences between these species. Nonetheless, in view of the hypothesis that mitochondria play a major role in mammalian ageing [33], these results point towards a putative role for oxidoreduction and mitochondrial alterations in the long lifespan of the naked mole-rat.

Although borderline significant, it is noteworthy that both fatty acid metabolism and lipid biosynthetic process functional groups figured among the top categories of over-expressed genes in naked mole-rats. According to Hulbert (2008), membrane fatty acid composition is correlated with the maximal lifespans of mammals through the reduction of oxidative damage caused by the autocatalytic ROS products of lipooxidation [34]. Another borderline significant category among over-expressed genes was endoplasmic reticulum, in which alterations have been suggested in naked mole-rat cells [35].

In conclusion, the largest effects on gene expression in comparing naked mole-rat and mouse liver RNAs suggest hypotheses about redox testable by genome engineering in the mouse. By employing a combination of Illumina/Solexa and 454 platforms for transcriptome sequencing, our differential gene expression analysis obviated the need for a reference naked mole-rat genome and paves the way for gene expression profiling of the naked mole-rat. Nonetheless, further studies of *Heterocephalus glaber*, an emerging model of successful ageing and cancer resistance, would greatly benefit from a reference genome sequence and we are currently developing efforts in this direction (http://www.naked-mole-rat.org/).

## Materials and Methods

### Library preparation and sequencing

Male young adult naked mole-rats and wild-derived mice (ID stock) were studied from colonies previously established [6], [7]. Specifically, wild-derived mice were roughly 6.5 months of age while 2 to 3 year-old naked mole-rats were used. Tissue harvesting from naked mole-rats was approved by the Institutional Animal Care and Use Committee (IACUC) at City College of New York (#0414 and #0415). Wild-derived mice samples were obtained under the University of Texas Health Science Center Institutional Animal Care & Use Committee approved Protocol #05002-34-05A.

In the first step, liver tissues from three naked mole-rats and three wild-derived house mice were mixed separately in similar quantities, and mRNA was extracted from the two samples with Dynabeads® mRNA DIRECT™ Kit (Invitrogen, USA). Full-length mRNA was then converted to double-strand, full-length cDNA with SuperScript® Double-Stranded cDNA Synthesis Kit (Invitrogen, USA), in which the oligo dT was replaced by the following primer: 5′-biotin-GAGCTGATTCTGGAGTTTTTTTTTTTTTTTTTTVN-3′ (BpmI recognizing site marked by underline, Agilent Technologies, USA). Exonuclease I (Epicenter, USA) digestion was then applied to remove biotin- BpmI-oligo-dT primer that did not participate in RT reaction and the products were then purified with QIAquick PCR Purification Kit (Qiagen, USA). In the second step, the purified double-strand, full-length cDNA was digested by NlaIII, and was then Qiagen purified. The resulted DNA-biotin complex was conjugated to M270 Dynabeads (Invitrogen, USA) and captured by a magnetic field. After being washed three times, the cDNA was released by being digested by BpmI, and was further Qiagen purified. In the third step, the resultant cDNA was blunted with End-It™ DNA End-Repair Kit (Epicenter, USA), and Qiagen purified. The libraries were then made according to the Illumina protocol and sequenced twice using the Illumina/Solexa platform, first with 39 bp single-end reads and then with 76 bp single-end reads.

The data, including the raw Illumina/Solexa reads, were deposited in the Gene Expression Omnibus (GEO) repository under accession GSE30764 (http://www.ncbi.nlm.nih.gov/geo/query/acc.cgi?acc=GSE30764). Our full results are also available online (http://www.naked-mole-rat.org/gene_expression.php).

### Naked mole-rat transcriptome assembly

Total RNA was isolated from naked mole-rat liver, brain and kidney using the RNeasy Blood and Tissue kit (Qiagen). Full length-enriched double-stranded cDNA was obtained using the Evrogen SMART kit. After nebulization, Roche 454 FLX libraries were prepared according to the manufacturer's protocols for genomic DNA. The 454 libraries were equipped with barcoded adapters [36], pooled and sequenced on one half of a 454 FLX sequencing plate, giving 350,671 sequences. An Illumina RNA-Seq library was prepared from kidney RNA according to the manufacturer's protocols. This library was sequenced on one lane of an Illumina GAIIx sequencer to yield 14.6 million 76-nt readings. All transcript data were assembled to contigs using the clc_novo_assemble program (CLC bio, Aarhus, Denmark) with default parameters. The raw mole-rat 454 data are available online (http://genome.imb-jena.de/mrat_blast/nmr_pool1.zip).

### Mapping wild-derived mouse reads to orthologous mouse genes

To map Solexa/Illumina 39 bp and 76 bp reads from wild-derived mouse liver, we constructed a reference library for mouse consisting of all mouse cDNA transcripts obtained from ENSEMBL (release 63). The reads were then mapped to this reference library with the short read mapper stampy (http://www.well.ox.ac.uk/project-stampy). All reads mapping outside of the 3′ end of the cDNA, i.e. 1050 upstream from the cDNA end, were discarded. After this step, 13.2 M out of the initial 24.3 M mouse 76 bp Solexa reads and 2.2 M out of 6.2 M mouse 39 bp Solexa reads were successfully mapped to the mRNA reads with positive mapping scores.

### Mapping naked mole-rat reads to mouse genes

Solexa/Illumina naked mole-rat reads were first mapped onto the naked mole-rat transcriptome contigs using stampy. Out of the 21.2 M 76 bp Solexa reads, 12.5 M reads (59.1%) mapped successfully including 12.2 M which had positive mapping scores while 4.1 M (44.7%) reads out of 9.2 M 39 bp Solexa reads with 3.4 M with positive mapping scores. The unaligned reads can be explained by the presence of many poly-T and poly-A reads generated by the PMAGE protocol which accounts for almost 50% of all the 39 bp Solexa reads.

To determine the mouse orthologous gene associated to each naked mole-rat read, we employed BLAST to map the contigs of the assembly onto mouse and guinea pig cDNAs. A combination of mouse and guinea pig transcriptomes was used because the annotation of the mouse genome is better than that of the guinea pig while the guinea pig genome is more similar to that of the naked mole-rat. The mappings of 39,402 contigs out of the initial 77,086 were judged to be unambiguous and only contigs mapping unambiguously were used. A mapping is unambiguous if its BLAST map is either unique and its e-value is less than 0.05 or it is 2 orders of magnitude smaller than the one with the second smallest. Mouse and guinea pig mappings were combined by checking if the contigs map to one to one orthologous genes in guinea pig and mouse. If a contig maps with lowest e-value to a guinea pig gene with no mouse ortholog or multiple mouse ortholog, the mapping is classified as ambiguous and all reads mapping to this contig are ignored in further analyses. For genes assigned to a guinea pig gene, the one to one orthologous mouse gene was then used in subsequent steps for the purpose of comparing naked mole-rat and mouse gene expression patterns. Ultimately, out of the 12.5 M reads mapped, 9.0 M could be assigned to an orthologous gene in the mouse reference genome. In sum, all contigs which map to either 1) mouse genes or 2) guinea pig with a one to one mouse ortholog, and with e-value at least 2 orders of magnitude smaller than the second lowest mapping e-value, are kept for counting naked mole-rat reads. The set of mouse genes these contigs map to is also the set of genes used as background for the functional analyses using DAVID.

### Normalization of read counts

Since two sequencing runs can have a different number of reads output and two samples can have different bias depending on how a library is prepared before sequencing, one needs to normalize the data before being able to tell whether a gene is differentially expressed in either samples. Many studies simply use as normalization the ratio of the output of the two sequencing runs, e.g. 21.2 M∶24.3 M in our case, or take into account read length for RNA-seq methods. However, it has been shown that these normalization methods may not work well when a sample has a different mRNA composition [37].

**Figure 3:**
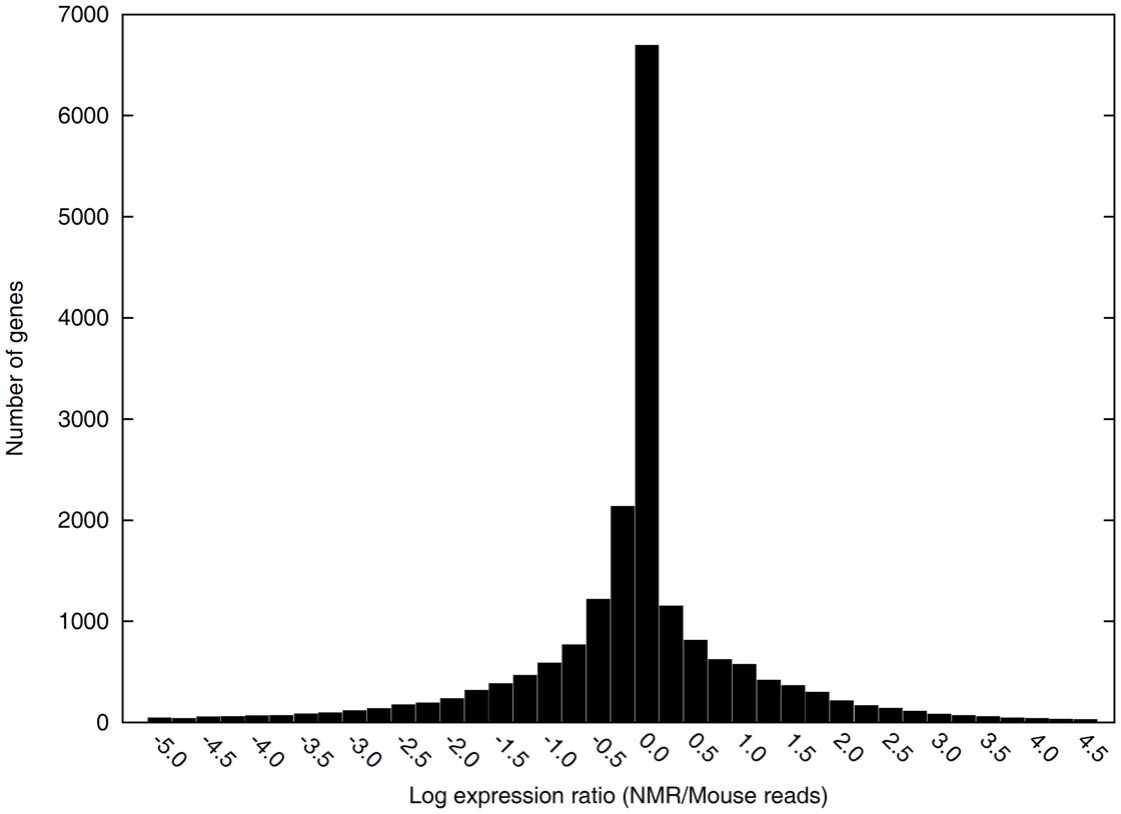
Histogram of the number of genes and their log expression ratio between naked mole-rat and wild-derived mouse reads. The ratio is computed with 100 pseudo-counts added to both naked mole-rat and wild-derived mouse reads.

In order to look at biases in our expression data, we plotted a histogram of the log expression level of every naked mole-rat gene to mouse genes and added 100 pseudo-counts to account for the large variance of the log expression difference of genes with low counts. As can be seen in Figure 3, the left tail of the histogram is much heavier than the right tail which we expect since there are many naked mole-rat Solexa reads that could not be mapped due to the imperfect coverage of our naked mole-rat contigs. However, the mode of the distribution being at 0 suggest the effect of other biases such as the size of sequencing output should not influence our estimation of differential fold expression as a significant normalization constant would translate into shifting the mode away significantly from 0.

### qPCR analysis

Tissue homogenisation of 100 mg of a single naked mole-rat liver was performed using a TissueLyser (Qiagen) for 3 minutes at 25 Hz. RNA was isolated by TRIzol/chloroform extraction using PureLink Mini Kit (Invitrogen) and exposed to on-column DNase I treatment according to the manufacturer's protocol. First strand cDNA synthesis was performed using 1 µg of total RNA, random primers and M-MLV reverse transcriptase (Invitrogen) according to the manufacturer's protocol.

Two microlitres of cDNA were amplified by PCR for the aforementioned transcripts under the following conditions: 2 minutes at 94°C, followed by 40 cycles of 30 seconds at 94°C, 30 seconds at 58°C, and 30 seconds at 72°C, followed by 2 minutes at 72°C. Primers were designed using the contigs that mapped with low e-values (e-value<10^−20^) to the orthologous genes and used primer3 [38] on the contigs. The following primers were used: *A2m*, 5′-GAACCGTCCTACCTCCAACA-3′ and 5′-TGTTGCTGACTTCAGTTCGG-3′; *Sat1*, 5′-TGGTATAGGATCAGAAATTTTGAAGA-3′ and 5′-TCCATCCCTCTTCACTGGAC-3′; *Sat2*, 5′-TCAAGGGATTGGTTCCAAAA-3′ and 5′-CAGGTGGGGACAGAGATGTT-5′; *Hprt1*, 5′-GCTTCCTTCTCCGCAGACT-3′ and 5′-CTTCATCACGTCTCGAGCAA-3′; *Tbp*, 5′-GAGAGGAGCTGCTTCGGATT-3′ and 5′-GCTCATGCCAGAGAATAGGC-3′; *Crym*, 5′-TCCCCCAAAGACTTGAACAC-3′ and 5′-CTGCCCTGAAAGAGTCTGGA-3′ . PCR products were electrophoresed on a 2% agarose gel to confirm specific amplification prior to qPCR analysis.

qPCR was performed in triplicate on the ABI 7500 RT-PCR System (Applied Biosystems) under the following conditions: 10 minutes at 95°C, followed by 40 cycles of 30 seconds at 95°C and 1 minute at 60°C. Reactions contained 12.5 µl Brilliant II SYBR Green QPCR Low Rox Master Mix (Agilent Technologies), 10.5 µl of cDNA, 1 µl of 5 µM forward primer and 1 µl of 5 µM reverse primer. Standard curves were generated for at least four different dilutions of cDNA to calculate the efficiency of qPCR reactions. Primer sets that generated an efficiency of between 1.93 and 2.07 and a correlation coefficient of less than −0.98 were selected. Analysis was performed similar to as was previously described [39]. Mean Ct values were calculated for those transcripts with selected primer sets. ΔCt was calculated as the difference between the mean Ct of Sat2 and the mean Ct of the selected transcript. Using the formula N_relative_ = E^ΔCt^, where E was the mean efficiency for all six transcripts, normalised expression levels relative to Sat2 were calculated. The number of Solexa reads per transcript was normalised to that of Sat2.

## Supporting Information

Dataset S1 **Full results from 76 bp Solexa run for all genes with at least one NMR read mapping to it.** The ratio is computed with 100 pseudo-counts added to both counts.
(XLS)

Dataset S2 **Full results for genes in GenAge database.** (http://genomics.senescence.info/genes/human.html).
(XLS)

Dataset S3 **Full results from DAVID enrichment analysis of genes over-expressed in the naked mole-rat.** Number at the end of worksheet name indicates fold change over-expression (5-, 10- and 15-fold over-expression) used as cutoff for selecting genes.
(XLS)
